# Anal metastasis of rectal cancer—adenocarcinoma of squamous cells: a case report and literature review

**DOI:** 10.1186/s40792-017-0319-x

**Published:** 2017-04-21

**Authors:** Shun Sasaki, Masahiko Sugiyama, Yu Nakaji, Ryota Nakanishi, Yuichiro Nakashima, Hiroshi Saeki, Eiji Oki, Yoshinao Oda, Yoshihiko Maehara

**Affiliations:** 1 Department of Surgery and Science, Graduate School of Medical Sciences, Kyushu University, 3-1-1 Maidashi, Higashi-ku, Fukuoka, 812-8582 Japan; 2 Department of Anatomic Pathology, Pathological Sciences, Graduate School of Medical Sciences, Kyushu University, Fukuoka, Japan

**Keywords:** Anal metastasis, Rectal cancer

## Abstract

Anal metastasis of colorectal cancer is very rare and is usually associated with a history of anal disease, including anal fistula, fissure, hemorrhoidectomy, and anastomotic injury. We report a case of rectal cancer with a synchronous anal metastasis consisting of adenocarcinoma of squamous cells without a history of anal disease. A 60-year-old woman had a chief complaint of melena. She had a 1.5-cm anal tumor on the perianal skin, and a Bollman type 2 rectal tumor on the Ra portion was found on colonoscopy. Biopsy of both tumors revealed a similar histology of well- to moderately differentiated adenocarcinoma. There was no sign of metastases in lymph nodes or other organs. For the purpose of diagnosis and treatment, transperineal local resection of the anal tumor was performed, and it was histologically identified as adenocarcinoma of squamous cells with no invasion to muscles, lymph ducts, or microvessels. The pathological margin was free. Then, to achieve radical cure, laparoscopic low anterior resection (LAR) with D3 lymphadenectomy was performed. The histological diagnosis of the anal tumor was adenocarcinoma of squamous cells without invasion to muscles, lymph ducts, or vessels. The surgical margin was completely free. Immunohistochemical analysis of both tumors revealed similar staining patterns, and the final diagnosis was rectal cancer with metastasis to the anal skin. The patient received no postoperative therapy, and no recurrences have been observed 12 months after surgery. We expect that our sphincter-preserving surgical strategy provided a good prognosis for the synchronous rectal cancer and anal metastasis. This is a rare report of a case with an anal metastasis of colorectal cancer on perianal squamous cells without a history of anal disease that was resected while preserving anal function.

## Background

Although many patients experience colorectal cancer metastasis, anal metastasis is very rare. Uncommonly, colorectal cancer can spread to the anus by lymphatic, hematogeneous, or peritoneal metastasis, or by direct extension. Yagi et al. reported that anal metastasis is observed in about 1% of all rectal carcinomas [1]. Treatment of anal metastasis has been controversial. Hsu et al. [2] stated that colorectal cancer metastases usually implant in the anus via epithelial damage from anal fistulas or fissures. Herein, we present a patient with synchronous rectal adenocarcinoma and anal metastasis on squamous cells in the absence of epithelial damage such as that due to fistulas or fissures. To the best of our knowledge, this is the first report in the literature of such an anal metastasis.

## Case presentation

**Fig. 1:**
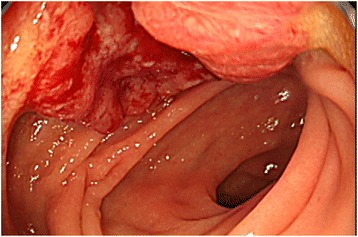
Colonoscopy image of a rectal carcinoma. The rectal carcinoma easily bled, with an ulcer around an irregular mass 10 cm from the anal verge. Biopsy revealed this was a well- to moderately differentiated carcinoma

**Fig. 2:**
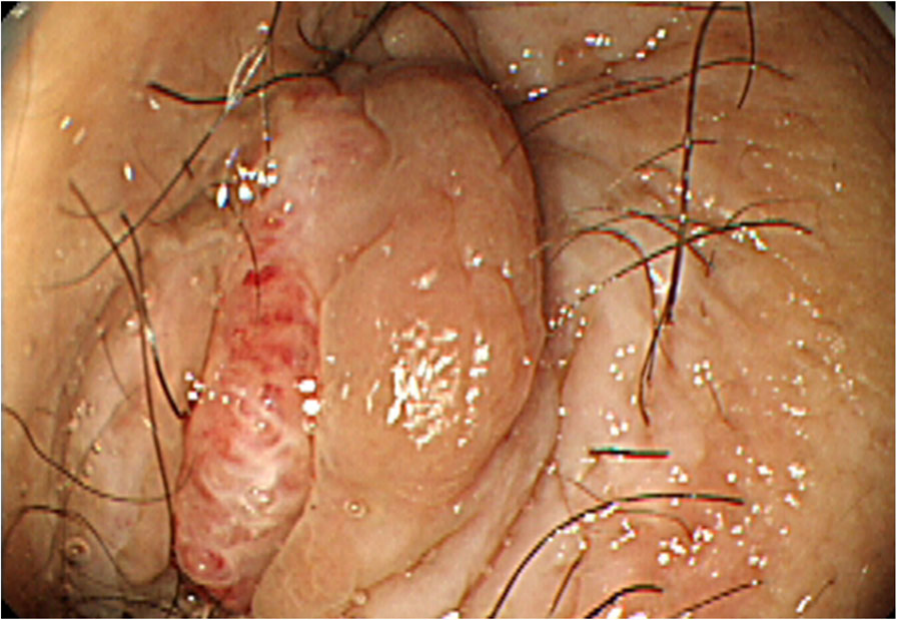
Colonoscopy image of an anal tumor. The tumor was a 1.0-cm irregular mass, displayed redness on the anal skin, and biopsy revealed the same histology as that of the rectal carcinoma

A 60-year-old woman without a significant medical history, including anal disease, presented with intermittent melena. Total colonoscopic examination showed a Bollman type 2 rectal tumor and a 1.5-cm anal tumor on the perianal skin. The rectal tumor easily bled 10 cm from the anal verge at the Ra portion (Fig. 1). The anal tumor had a diameter of 1 cm and was irregularly shaped and slightly red on the outside of the dentate line (Fig. 2). Biopsy of both lesions revealed moderately differentiated carcinoma. Computed tomography (CT), including positron emission tomography-CT (PET-CT) and magnetic resonance imaging showed no evidence of other local invasion or metastases. The carcinoembryonic antigen (CEA) level was elevated at 18.2 ng/mL; our institutional normal range is 0–3.6 ng/mL. For diagnostic and therapeutic purposes, anal tumor resection was performed. The tumor was resected along with a portion of the internal anal sphincter muscle. Pathological diagnosis of the anal tumor was well- to moderately differentiated adenocarcinoma, and the tumor had developed in the squamous epithelial layer with no evidence of inflammation or fibrosis of anal fistulas or fissures. The pathological margin was free, and no lymph duct invasion (ly0) or vessel invasion (v0) was observed. There were no postoperative complications, and the patient was discharged from our hospital 6 days after surgery. The tumor was clinically diagnosed as perianal metastasis.

**Fig. 3:**
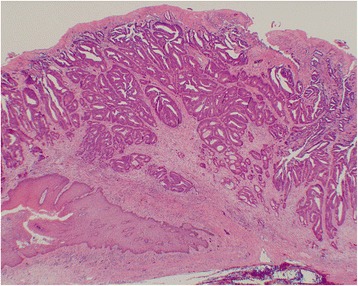
The anal tumor stained with hematoxylin and eosin. Moderately differentiated adenocarcinoma was observed from the squamous epithelium to the submucosal tissue

**Fig. 4:**
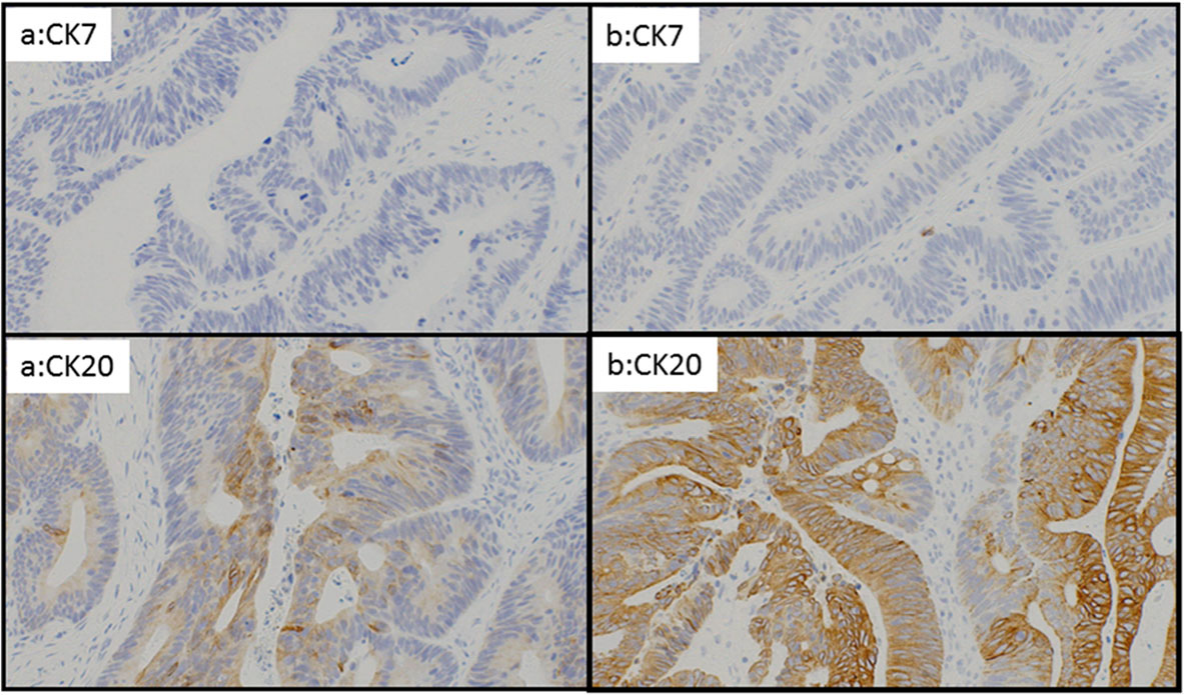
Immunohistochemical staining of the rectal (**a**) and anal (**b**) carcinomas with cytokeratin (CK) 7 and 20. Both tumors were CK7-negative and CK20-positive

Subsequently, the patient underwent laparoscopic lower anterior resection (LAR) and D3 lymphadenectomy to excise the rectal tumor. This tumor had the same pathology as the anal tumor, well- to moderately differentiated adenocarcinoma, and the cytokeratin (CK) 7 and CK20 immunohistochemical staining patterns were also identical: CK7 was negative in both the rectal and anal the adenocarcinomas, while CK20 was positive in both tumors (Figs. 3 and 4). The rectal tumor penetrated to the surface of the visceral peritoneum (T4a). It exhibited vessel invasion (v2), but no lymph duct invasion (ly0). The surgical margin was free, and there was no continuity with the anal tumor. No metastases were observed in lymph nodes (N0). Ten days after surgery, the patient had no postoperative complications and left our hospital. The rectal carcinoma was finally diagnosed as perianal metastasis. Since radical resection was performed, no adjuvant chemotherapy has been administered. There is no sign of recurrence 12 months after surgery.

### Discussion

Although many colorectal cancer patients develop metastases, anal metastasis is unusual. Moreover, rectal carcinoma with synchronous anal metastasis is extremely rare [3], is difficult to diagnose, and no standardized therapeutic strategy exists [4]. In the present case, we performed local resection of the anal tumor to determine whether it was a primary cancer or represented metastasis of the rectal carcinoma. The anal tumor was relatively soft and mobile. We estimated that it was shallow and resectable. We considered two situations: if the anal tumor was a metastasis of the rectal cancer and no other distant metastases were found, resection of the metastasis and the primary rectal tumor could be curative. If the anal tumor was the primary cancer, LAR of the rectal cancer would also be curative. If the tumor exhibited deep invasion, we might have performed more extensive surgery, such as abdominoperineal resection (APR). Pathological findings revealed that the anal tumor was limited to the squamous cells of the perianal skin, lacking continuity with the rectal tumor. Therefore, laparoscopic LAR rather than APR was used to remove the rectal carcinoma with curative intent. We were able to preserve the anal sphincter. Immunohistochemical analysis was the key for diagnosing the anal metastasis of the rectal cancer. As no significant evidence supports the efficacy of adjuvant chemotherapy and radiotherapy for stage IV colorectal patients who underwent resection of the primary and metastatic tumors, adjuvant chemotherapy was not administered.

Several reports have indicated that immunohistochemical analysis is critical for confirming a diagnosis of anal metastasis of rectal carcinoma [3, 5–7]. Cytokeratin (CK) is an epithelial protein, so determination of its presence is helpful for establishing the origin of metastatic carcinoma [8]. In particular, CK7 and CK20 are useful for detecting primary epithelial tumors: CK7 negativity and CK20 positivity are characteristic of colorectal cancer.

We performed a literature search in PubMed using the terms “synchronous”, “anal metastasis”, and “colorectal cancer” from 2000 to 2014. Only 18 cases have been previously documented. The clinicopathological profiles of these 18 cases, as well as of the present case, are summarized in Table 1. Almost all of the cases had a history of anal fistulas or fissures. The optimal surgical procedure for colorectal cancer with anal metastasis has been controversial [4, 6, 7]. For the purpose of radical resection, APR has been performed in some cases, particularly in Japan. Chemotherapy and radiation therapy for colorectal cancer have enabled preservation of anal function in other cases. The reports of Hamada [6] and Ishiyama [9] showed that anal metastasis of colorectal cancer could be treated with sphincter-preserving surgery. When local resection is selected for anal metastasis, it is critical that the surgical margin is free of tumor cells to prevent local recurrence. If the anal tumor is so wide that local resection with adequate margins is difficult, radiation therapy is likely beneficial, as observed in the case of Benjelloun [7]. As local resection is recommended for liver or lung metastasis of colorectal carcinoma, local resection of anal metastasis may also be beneficial [6]. Further evaluation of treatments for anal metastasis of colorectal cancer, which might include chemotherapy and radiation therapy, is required.

**Table 1:** Reports of synchronous anal metastasis of colorectal cancer

Author	Age/gender	Primary tumor	T	N	History of anal injury	Therapy	Prognosis
Tokuhara [19]	69/M	S	Ss	0	+	APR	NA
Yoshimura [20]	59/M	Ra	Ss	1	+	APR→CT	43M
Shinohara [21]	36/M	Ra	Ss	1	+	LAR→LR	6M
Shimoyama [22]	61/M	S	Ss	1	+	APR	60M
Hyman [5]	66/M	S	Ss	1	+	APR	12M
Mizutani [15]	63/F	Ra	A	1	−	PE→CT	21M
Narita [16]	72/M	Rs	Si	1	−	APR→CT	NA
Zbar [23]	54/M	S	NA	NA	+	HAR+LR→CRT	14M
Hamada [6]	61/M	S	mp	0	+	LAR→CT	12M
Gupta [24]	44/M	D	Ss	1	+	CR+LR→CT	36M
Ishiyama [9]	53/M	Ra	Ss	1	+	LAR→LR	10M
Sandiford [3]	72/M	Rs	NA	0	+	LAR+LR→CRT	14M
Morita [19]	61/M	S	Ss	2	−	LR→Hartmann→CT	7M
Godai [25]	60/M	Rb	A	1	+	APR→CT	28M
Watanabe [21]	68/M	Rs	Se	1	−	APR	8M
Benjelloun [7]	68/M	Rs	mp	0	+	CRT→HAR+LR	36M
Benjelloun [7]	55/M	Rs	Ss	0	−	CRT→HAR+LR	36M
Gomes [26]	65/M	S	Ss	0	+	APR+CR	3M
Present case	60/F	Ra	Ss	0	−	LR→LAR	12M

Anal metastasis from colorectal carcinoma may occur in various ways [2]; hematogenous spread or implantation were both possible in the present case. If metastasis occurred via implantation, local excision of the anal tumor and LAR with sphincter preservation are possible means of radical resection [4]. Malignant cells are thought to implant on injured anal tissue [10], which may occur following treatment of an anal fistula or anastomosis formation during rectal surgery. Since cancer cells were present from the squamous epithelium to the submucosal tissue of the anus, implantation was possible. However, due to the lack of a history of anal injuries and invasion of tumor cells to vessels, hematogenous spread must also be considered. Tumor invasion to lymphatic ducts and vessels is present in most cases of anal metastasis of colorectal cancer without an anal injury history [11–14]. These reports suggest that obstruction of the upper lymphatic and hematogenous streams spreads cancer cells toward the anal region, thereby leading to anal metastasis.

In this case, no adjuvant chemotherapy was used based on the Japanese guideline for the treatment of colorectal liver metastases (CRLM), Clinical Question #8 (CQ8) [15]. Some studies have shown that adjuvant chemotherapy for CRLM can improve relapse-free survival and disease-free survival, but not overall survival [15–17]. However, other research has suggested that adjuvant chemotherapy for CRLM does improve overall survival, and that it should be administered based on risk factors, which include diabetes mellitus, depth and number of lymph node metastases of the primary cancer, number and size of liver metastases, and time to recurrence [18]. In the present case, the rectal tumor penetrated the surface of the visceral peritoneum (T4a), but no other risk factors were present. Adjuvant chemotherapy may therefore have had little benefit. For distant colorectal cancer metastasis, particularly anal metastasis, no evidence of efficacy of adjuvant chemotherapy exists; thus, effective treatment strategies should be explored. The present patient should be closely monitored for signs of recurrence.

## Conclusions

We experienced a rare case of synchronous rectal adenocarcinoma and anal metastasis of squamous cells in the absence of epithelial damage due to fistulas or fissures. Immunohistochemical analysis appears to have been crucial for confirming this diagnosis, which enabled sphincter-preserving surgery to be performed.

## Abbreviations

APR: Abdominoperineal resection
CR: Colon resection
CRT: Chemoradiation therapy
CT: Chemotherapy
D: Descending colon
F: Female
HAR: High anterior resection
LAR: Low anterior resection
LR: Local resection
M: Male
NA: Not available
PE: Pelvic exenteration
R: Rectal
S: Sigmoid colon
